# Few‐Atomic‐Layered Co‐Doped BiOBr Nanosheet: Free‐Standing Anode with Ultrahigh Mass Loading for “Rocking Chair” Zinc‐Ion Battery

**DOI:** 10.1002/advs.202204087

**Published:** 2022-09-13

**Authors:** Bei Long, Qing Zhang, Tengfei Duan, Ting Song, Yong Pei, Xianyou Wang, Chunyi Zhi, Xiongwei Wu, Qianyu Zhang, Yuping Wu

**Affiliations:** ^1^ School of Chemistry Xiangtan University Xiangtan 411105 P. R. China; ^2^ Department of Materials Science and Engineering City University of Hong Kong Hong Kong 999077 P. R. China; ^3^ School of Chemistry and Materials Science Hunan Agricultural University Changsha 410128 P. R. China; ^4^ College of Materials Science and Engineering Sichuan University Chengdu Sichuan 610064 P. R. China; ^5^ School of Energy and Environment Southeast University Nanjing 211189 P. R. China

**Keywords:** Co‐doped BiOBr, few‐atomic‐layered nanosheets, insertion‐conversion mechanism, long cyclic life, ultrahigh mass loading

## Abstract

Insertion host materials are considered as a candidate to replace metallic Zn anode. However, the high mass loading anode with good electrochemical performances is reported rarely. Herein, a few‐atomic‐layered Co‐doped BiOBr nanosheet (Co‐UTBiOBr) is prepared via one‐step hydrothermal method and a free‐standing flexible electrode consisting of Co‐UTBiOBr and CNTs is designed. Ultrathin nanosheet (3 atomic layers) and CNTs accelerate Zn^2+^ and electron transfer respectively. The Co‐doping is conducive to the reduced Zn^2+^ diffusion barrier, the improved volume expansion after Zn^2+^ intercalation, and the enhanced electronic conductivity of BiOBr, verified by experimental and theoretical studies. An insertion‐conversion mechanism is proposed according to ex situ characterizations. Benefiting from many advantages, Co‐UTBiOBr displays a high capacity of 150 mAh g^−1^ at 0.1 A g^−1^ and a long‐term cyclic life with ≈100% capacity attention over 3000 cycles at 1 A g^−1^. Remarkably, excellent electrochemical performances are maintained even at an ultrahigh mass loading of 15 mg cm^−2^. Co‐UTBiOBr//MnO_2_ “rocking chair” zinc‐ion battery exhibits a stable capacity of ≈130 mAh g^−1^ at 0.2 A g^−1^ during cyclic test and its flexible quasi‐solid‐state battery shows outstanding stability under various bending states. This work provides a new idea for designing high mass loading anode.

## Graphical abstract

Few‐atomic‐layered Co‐doped BiOBr nanosheet is prepared as anode of zinc‐ion batteries. Ultrathin nanosheet and Co‐doping improve charge transfer and volume change, proved via experimental and theoretical studies. Ex situ tests reveal the insertion‐conversion mechanism of UTBiOBr. Co‐UTBiOBr displays satisfactory electrochemical performances even at ultrahigh mass loading of 15 mg cm^−2^. Significantly, Co‐UTBiOBr//MnO_2_ “rocking chair” zinc‐ion battery shows good cyclic stability.

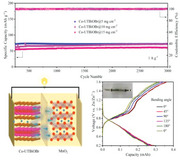

## 1 Introduction

Zinc‐ion batteries (ZIBs) have drawn the attention from researchers due to their high theoretical capacity (825 mAh g^−1^ and 5854 mAh cm^−3^), inherent safety, and economic feasibility.^[^ ^1^, ^2^, ^3^, ^4^, ^5^, ^6^, ^7^, ^8^ ^]^ Up to now, a great advance has been made in the study of aqueous ZIBs. Abundant Mn‐based and V‐based materials have been developed as cathodes of ZIBs and they show excellent electrochemical properties and a wide application perspective.^[^ ^9^, ^10^, ^11^, ^12^, ^13^, ^14^ ^]^ However, the utilization of metallic Zn anode is impeded by Zn dendrite, dead Zn, byproduct formation, and water consumption.^[^ ^15^, ^16^, ^17^, ^18^, ^19^ ^]^ On the one hand, many methods have been tried to improve metallic Zn anode, such as artificial solid/electrolyte interphase layers, electrolyte additives, and Zn composite anodes.^[^ ^20^, ^21^, ^22^, ^23^ ^]^ On the other hand, more and more insertion host materials are used as anodes of “rocking chair” ZIBs due to their high capacities, low discharge platforms, and dendrite‐free operation.^[^ ^24^, ^25^, ^26^, ^27^ ^]^

Among the reported metal compound anodes, Ti/Cu/Mo‐based materials exhibit good electrochemical properties.^[^ ^28^, ^29^, ^30^, ^31^, ^32^, ^33^ ^]^ Li and coworkers reported a presodiated TiS_2_ and it showed a capacity of 140 mAh g^−1^ with a suitable potential of 0.3 V (vs Zn^2+^/Zn) at 0.05 A g^−1^ and a good cyclability of 77% retention over 5000 cycles at 0.5 A g^−1^.^[^ ^28^ ^]^ Zhang et al. designed a periodically stacked CuS‐CTAB superlattice which showed a low discharge plateau of 0.4 V (vs Zn^2+^/Zn), a good rate performance of 225.3/144.4 mAh g^−1^ at 0.1/10 A g^−1^, and a superior cyclability of 87.6% retention over 3400 cycles at 10 A g^−1^.^[^ ^29^ ^]^ Xiong et al. used hexagonal MoO_3_ as an intercalation anode with an average discharge voltage of 0.35 V (vs Zn^2+^/Zn) and it exhibited a capacity of 120 mAh g^−1^ at 0.2 A g^−1^ and a negligible capacity fading after 100 cycles at 0.3 A g^−1^.^[^ ^30^ ^]^ It may seem that we have found suitable insertion host material to design “rocking chair” ZIBs. However, the mass loading of most reported anodes (≤5 mg cm^−2^) is low and their battery performances cannot reflect real performances in practical applications.^[^ ^28^, ^29^, ^30^, ^34^, ^35^, ^36^ ^]^ The rate capability and cyclic life of the high mass loading electrode are often much lower than those of the low mass loading electrode, resulting from poor charge transfer and electrode design.^[^ ^34^, ^35^, ^36^ ^]^ Thus, it is very important to design high mass loading anode with satisfactory electrochemical activity.

Now, some high mass loading cathodes have been designed using different methods. For example, H_11_Al_2_V_6_O_23.2_@graphene, Mg_0.19_V_2_O_5_ 0.99H_2_O, and MXene@MnO_2_ cathodes with high mass loading are gained by simply increasing the thicknesses of the coating films on current collector.^[^ ^34^, ^35^, ^36^ ^]^ To maintain the superior electrochemical properties of electrode with high mass loading, this method requires a material with fast charge transfer and good structural stability. In addition, some 3D substrates with good conductivity, such as carbon‐fiber paper and stainless steel mesh, are used to construct high mass loading cathodes and the large contact area between 3D substrate and active material favors the charge transport and structural stability of the electrode.^[^ ^37^, ^38^ ^]^ Unfortunately, there is a rare report on a high mass loading anode. Thus, the design and study of high mass loading anode are necessary to promote the development of “rocking chair” ZIBs.

With a special layered structure, BiOXs (X = Cl, Br, I) are considered as potential electrodes in ZIBs. BiOI with a low diffusion barrier (0.57 eV) shows good rate performances but slow capacity decay during cyclic test and the battery performances of high mass electrodes are not studied.^[^ ^8^ ^]^ BiOBr with good chemical durability also has great potential for application in ZIBs, but its large diffusion barrier (1.06 eV) needs to be improved.^[^ ^8^ ^]^ In this work, Co‐doped BiOBr ultrathin nanosheet (Co‐UTBiOBr) is synthesized by one‐step hydrothermal method and used as insertion host material. The free‐standing nanopaper electrode consists of Co‐UTBiOBr and CNTs. The utilization of CNTs and the design of ultrathin nanosheets prompt the fast transfer of electrons and zinc ions during electrochemical tests. The slight Co doping reduces the Zn^2+^ diffusion barrier, increases the electronic conductivity of BiOBr, and inhibits the volume change of BiOBr after Zn^2+^ intercalation, as proved by density functional theory (DFT) calculation and experimental study. The insertion‐conversion mechanism of BiOBr is proved by ex situ tests. The Co‐UTBiOBr‐based nanopaper electrode with superior charge transfer shows excellent rate performance and cyclic stability even with an ultrahigh mass loading of 15 mg cm^−2^. Moreover, Co‐UTBiOBr//MnO_2_ “rocking chair” ZIB shows a high discharge capacity and the self‐assembled flexible battery displays excellent stability during flexible electrochemical tests.

## 2 Results and Discussion

**Figure 1:**
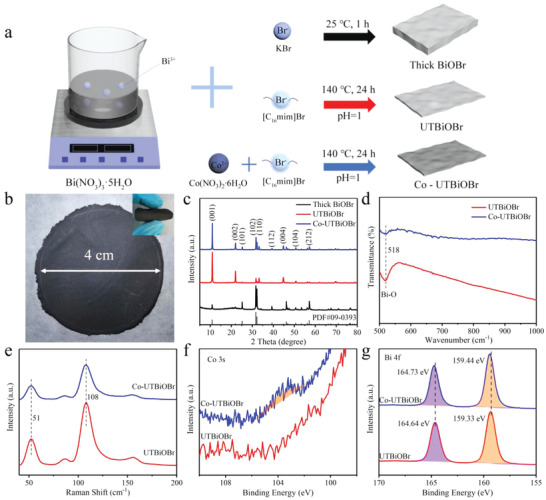
a) Schematic diagram of the preparation of thick BiOBr, UTBiOBr, and Co‐UTBiOBr. b) Digital photographs of free‐standing flexible electrodes. c) XRD patterns of thick BiOBr, UTBiOBr, and Co‐UTBiOBr. d) FT‐IR, e) Raman, f) Co 3s XPS, and g) Bi 4f XPS spectra of UTBiOBr and Co‐UTBiOBr.

Schematic diagram of the preparation of BiOBr‐based materials is displayed in **Figure** **1**a. The thick BiOBr is prepared by a soft chemistry method employing KBr as Br source. The BiOBr ultrathin nanosheet is obtained by using [C_16_mim]Br to replace KBr. The Br^−^ in [C_16_mim]Br as a capping agent is slowly released to react with Bi^3+^ in the preparation process, which results in a decreased reaction rate. Furthermore, the BiOBr core is covered by C_16_mim and the C_16_ long carbon chain restrains the further stacking and growth along the (001) direction, which generates ultrathin nanosheet. Co‐UTBiOBr is prepared via adding a certain amount of Co(NO_3_)_2_∙6H_2_O to the reaction system and some Bi^3+^ will be replaced by Co^2+^. Free‐standing BiOBr‐based electrodes with good flexibility are obtained by vacuum suction filtration of the suspension consisting of active materials and CNTs (Figure 1b).

To prove the successful preparation of target material, many characterizations are performed. In the X‐ray diffraction (XRD) patterns the characteristic peaks of thick BiOBr match with the standard card of tetragonal BiOBr (PDF#09‐0393) and its peak of (001) plane is relatively weak (Figure 1c). While UTBiOBr and Co‐UTBiOBr display the visibly strong (001) peak and there is no impurity peak in Co‐UTBiOBr. In the Fourier transform infrared (FT‐IR) spectra the absorption peak at 518 cm^−1^ is ascribed to the Bi—O stretching mode (Figure 1d).^[^ ^39^, ^40^ ^]^ And Raman peaks at 51 and 108 cm^−1^ match with the Bi—Br stretching modes of BiOBr, as seen in Figure 1e.^[^ ^39^, ^40^ ^]^ The successful Co‐doping in Co‐UTBiOBr is proved by XPS spectra. Co‐UTBiOBr shows a weak peak and there is no obvious signal for UTBiOBr in their Co 3s XPS spectra (Figure 1f). The result of ICP‐OES indicates that the ratio of Co : Bi in Co‐UTBiOBr is 0.056 : 1. Therefore, 93.3% of Co in raw material (0.06 mmol Co(NO_3_)_2_∙6H_2_O) is incorporated into the BiOBr nanosheets. The Co replaces Bi and is bonded with O in the Co‐doped UTBiOBr, which is consistent with previous research.^[^ ^41^, ^42^ ^]^ Moreover, the Bi 4f_5/2_ and Bi 4f_7/2_ peaks of Co‐UTBiOBr shift to higher binding energy compared to those of UTBiOBr, indicating the increased valence state of bismuth ion in Co‐UTBiOBr (Figure 1 g). The formation of Bi^(3+x)+^ is to prevent charge imbalance caused by the introduction of Co^2+^. The above analyses prove the successful preparation of BiOBr and Co‐doped BiOBr.

**Figure 2:**
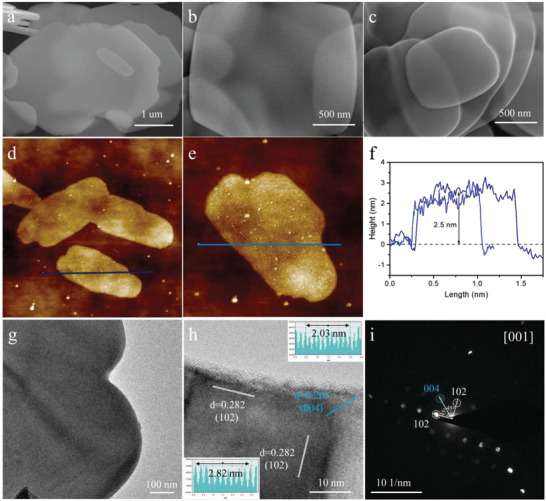
SEM images of a) thick BiOBr, b) UTBiOBr, and c) Co‐UTBiOBr. d,e) AFM images and f) their corresponding thickness measurements of Co‐UTBiOBr. g) TEM, h) HRTEM, and i) SAED images of Co‐UTBiOBr.

The morphology of materials is observed via scanning electron microscope (SEM), atomic force microscope (AFM), and transmission electron microscope (TEM) images. Obviously, three kinds of BiOBr are flake structures with smooth surface, as depicted in **Figure** **2**a–c and Figure S1, Supporting Information. But the thicknesses of UTBiOBr and Co‐UTBiOBr are obviously smaller than that of thick BiOBr and their thicknesses are measured by AFM. The thicknesses of UTBiOBr and Co‐UTBiOBr are about 3.5 and 2.6 nm, reflecting their ultrathin nanosheet structures and much less than that of thick BiOBr (18 nm) (Figure 2d–f, Figures S2 and S3, Supporting Information). The thickness of BiOBr monoatomic layer is about 0.89 nm, which reveals that thick BiOBr, UTBiOBr, and Co‐UTBiOBr are composed of about 20, 4, and 3 atomic layers respectively. The ultrathin nanosheet shortens the distance of Zn^2+^ diffusion and therefore the fast Zn^2+^ transfer and storage. The flake structure of Co‐UTBiOBr is observed by the TEM image further and its high‐resolution TEM (HRTEM) image shows clear crystal fringes (Figure 2g,h). Two sets of lattice fringes with a lattice spacing of 0.282 nm correspond to the (102) facet of BiOBr, and the lattice distance of 0.203 nm is assigned to the (004) plane of BiOBr. The corresponding selected area electron diffraction (SAED) spectrum reflects the single crystal structure (Figure 2i). The angle of (102)/(004) and (102) facets is 90°/45°, indicating that the exposed facet of this nanosheet is (001).^[^ ^43^ ^]^ This also explains the strong (001) peaks of UTBiOBr and Co‐UTBiOBr in XRD patterns. The element mapping reveals the uniform Co‐doping (Figure S4, Supporting Information). The nitrogen adsorption/desorption isotherms are exhibited in Figure S5, Supporting Information. The nearly overlapping nitrogen adsorption and desorption curves of BiOBr result from its almost nonporous characteristic. The specific surface area of Co‐UTBiOBr (2.34 m^2^ g^−1^) is slightly greater than that of UTBiOBr (0.54 m^2^ g^−1^) due to the smaller thickness of Co‐UTBiOBr. The above results prove the successful design of a few‐atomic‐layered Co‐doped BiOBr nanosheet. The free‐standing Co‐UTBiOBr electrodes with different mass loading are also observed. As displayed in Figure S6a1–c1, Supporting Information, the porous nature and 3D interwoven architecture of free‐standing Co‐UTBiOBr electrodes can be easily identified and CNTs effectively prevent the self‐stacking of Co‐UTBiOBr. Figure S6a2–c2, Supporting Information, exhibits the cross‐sectional images of free‐standing Co‐UTBiOBr electrodes with different mass loading. As the increasing mass loading, the thicknesses of electrodes increase. The average thicknesses of free‐standing Co‐UTBiOBr electrodes with mass loading of 5, 10, and 15 mg cm^−2^ are 0.12, 0.23, and 0.34 mm respectively. As shown in enlarged cross‐sectional SEM images (Figure S6a3–c3, Supporting Information), Co‐UTBiOBr are well‐dispersed across the cross‐linked CNTs conductive network and well wrapped by CNTs.

The electrochemical properties of materials in half cells are compared further. The cyclic voltammetry (CV) curves are tested first, as shown in Figure S7, Supporting Information. The obvious redox peaks indicate Zn^2+^ intercalation and deintercalation and their Zn^2+^ storage mechanism will be studied by ex situ tests. Three electrodes show similar CV curves, suggesting their alike energy storage mechanism. The difference is that the reduction and oxidation peaks of Co‐UTBiOBr shift to higher and lower potentials in comparison with thick BiOBr and UTBiOBr, revealing that both designs of few atomic layers and Co‐doping are beneficial to the decreased polarization. The ultrathin structure can shorten the distance of Zn^2+^ diffusion, which has been studied in previous reports.^[^ ^44^, ^45^ ^]^ The Co doping decreases Zn^2+^ diffusion barrier, which will be proved in the subsequent tests and theoretical calculations. Galvanostatic charge‐discharge (GCD) curves of electrodes are analyzed further (Figure S8, Supporting Information). They also show similar shapes, which is consistent with the results of CV curves. Their average voltages in the discharge processes are about 0.4 V, indicating that BiOBr‐based materials are appropriate anodes. Furthermore, the potential gap of Co‐UTBiOBr (0.08 V) is lower than those of thick BiOBr (0.12 V) and UTBiOBr (0.11 V), also reflecting its elevated electrochemical reaction kinetics.

**Figure 3:**
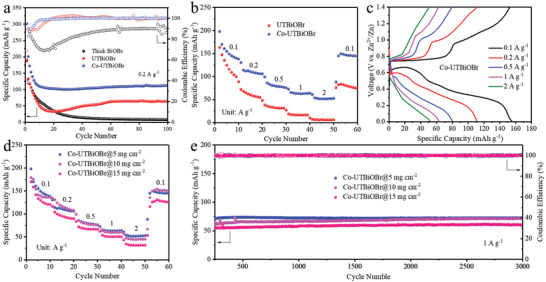
a) Cyclic tests at 0.2 A g^−1^ of thick BiOBr, UTBiOBr, and Co‐UTBiOBr with mass loading of 5 mg cm^−2^. b) Rate capabilities of UTBiOBr and Co‐UTBiOBr with mass loading of 5 mg cm^−2^. c) GCD curves at different current densities of Co‐UTBiOBr with mass loading of 5 mg cm^−2^. d) Rate and e) cycle performances of Co‐UTBiOBr with mass loading of 5, 10, and 15 mg cm^−2^.

The cyclic life at 0.2 A g^−1^ of thick BiOBr, UTBiOBr, and Co‐UTBiOBr with mass loading of 5 mg cm^−2^ is tested to preliminarily reveal their structural stability (**Figure** **3**a). It is obvious that thick BiOBr shows a fast capacity fading and low coulombic efficiencies and its discharge capacity is 8 mAh g^−1^ after 100 cycles, suggesting its poor structural stability during Zn^2+^ insertion/deinsertion. The XRD pattern of thick BiOBr after 1 cycle indicates that it suffers from a high level of irreversible reaction (Figure S9, Supporting Information). By contrast, UTBiOBr shows a relatively stable charge‐discharge process after the initial capacity decay and high coulombic efficiencies as well as delivers a specific capacity of 64 mAh g^−1^ after 100 cycles, demonstrating that the ultrathin nanosheet structure is beneficial to the electrochemical reversibility of Zn^2+^ storage. The further Co‐doping leads to higher coulombic efficiencies and capacity retention (100 mAh g^−1^ after 100 cycles) of Co‐UTBiOBr, revealing the benefit of Co‐doping. In situ electrochemical impedance spectra (EIS) are recorded to evaluate the structural stability of UTBiOBr and Co‐UTBiOBr (Figure S10, Supporting Information). Their resistance values are close during cyclic tests, but the resistance variation of Co‐UTBiOBr is lower than that of UTBiOBr, also suggesting the better structural stability of Co‐UTBiOBr (Tables S1 and S2, Supporting Information). In addition, the rate performances show the superiority of Co‐UTBiOBr (Figure 3b). At the current densities of 0.1, 0.2, 0.5, 1, and 2 A g^−1^, the discharge capacities of Co‐UTBiOBr are 150, 110, 80, 63, and 52 mAh g^−1^, which is distinctly higher than those of UTBiOBr (125, 60, 33, 17, and 7 mAh g^−1^). When the current returns back to 0.1 A g^−1^, the discharge capacities of UTBiOBr and Co‐UTBiOBr are 71 and 147 mAh g^−1^. Their GCD curves at different current densities are compared (Figure 3c and Figure S11, Supporting Information). Compared to UTBiOBr, GCD curves of Co‐UTBiOBr show more obvious charge and discharge plateaus at large current density. The cyclic life of 0.04Co‐UTBiOBr, Co‐UTBiOBr (0.06Co‐UTBiOBr), and 0.08Co‐UTBiOBr is exhibited in Figure S12, Supporting Information, demonstrating that a proper amount of Co doping is very important. The above results indicate the fast reaction dynamics and high electrochemical reversibility of Co‐UTBiOBr, which encourages us to further explore the battery performances of Co‐UTBiOBr with commercial‐level mass loading.

The electrochemical properties of Co‐UTBiOBr with mass loading of 5, 10, and 15 mg cm^−2^ are compared to reveal its potential practical application. Their 1st–3rd GCD curves show similar shape and same potential gaps, reflecting the outstanding electrochemical reaction kinetics of Co‐UTBiOBr (Figures S8c and S13, Supporting Information). The rate capability of Co‐UTBiOBr (140, 115, 78, 60, and 45 mAh g^−1^) with a mass loading of 10 mg cm^−2^ is quite close to that of the electrode with a mass loading of 5 mg cm^−2^ (Figure 3d). Even with an ultrahigh mass loading of 15 mg cm^−2^ Co‐UTBiOBr still shows satisfactory rate capability of 130, 94, 67, 51, and 33 mAh g^−1^. Their GCD curves at different current densities are analyzed (Figure 3c and Figure S14, Supporting Information). Co‐UTBiOBr with mass loading of 10 and 15 mg cm^−2^ still shows visible charge and discharge plateaus at 1 A g^−1^. To further demonstrate the superiority of Co‐UTBiOBr, the cyclic life is tested at the current density of 1 A g^−1^ (Figure 3e). The discharge‐specific capacities of Co‐UTBiOBr with mass loading of 5, 10, and 15 mg cm^−2^ are 73, 71, and 61 mAh g^−1^ after 3000 cycles and their ≈100% capacity attentions are obtained. All three exhibit overlapping charge and discharge curves during cyclic tests (Figure S15, Supporting Information). To highlight the superiority of Co‐UTBiOBr, its battery performances are compared to reported anodes, as displayed in Table S3, Supporting Information. The mass loading of most anodes is < 3 mg cm^−2^ and no high mass loading anode (≥ 10 mg cm^−2^) is reported. Meanwhile, the cyclic stability of our electrode is superior to most reported anodes. All of these reveal its extremely high electrochemical stability and great potential for application.

To analyze the electrochemical kinetics of UTBiOBr and Co‐UTBiOBr, CV curves from 0.1 to 1.0 mV s^−1^ are recorded in Figure S16, Supporting Information, and relevant details and calculation formulas are supplied in experimental methods. The b values are gained by counting the slopes of the log(i) versus log(v) plots, as exhibited in Figure S17, Supporting Information. Both show large b values (0.74–0.87), indicating their dominant pseudocapacitive behaviors. Moreover, *b* values of Co‐UTBiOBr are bigger than those of UTBiOBr. The accurate pseudocapacitive and diffusion‐controlled contributions of electrodes are calculated further. The pseudocapacitive contributions of UTBiOBr and Co‐UTBiOBr at a low scan rate of 0.1 mV s^−1^ are 59.4% and 69.3% and the ratios gradually increase with the increment of scan rates (Figure S18, Supporting Information). 83.4% of the total capacity of Co‐UTBiOBr is attributed to the surface capacitance process at 1 mV s^−1^, as seen in Figure S19, Supporting Information. Furthermore, the Zn^2+^ diffusion coefficients of electrodes are obtained according to galvanostatic intermittent titration technique (GITT) curves and Fick's second law (Figure S20, Supporting Information). Obviously, the Zn^2+^ diffusion coefficients of Co‐UTBiOBr are larger than those of UTBiOBr. The above analyses prove that Co‐doping accelerates the transfer and storage of Zn^2+^.

**Figure 4:**
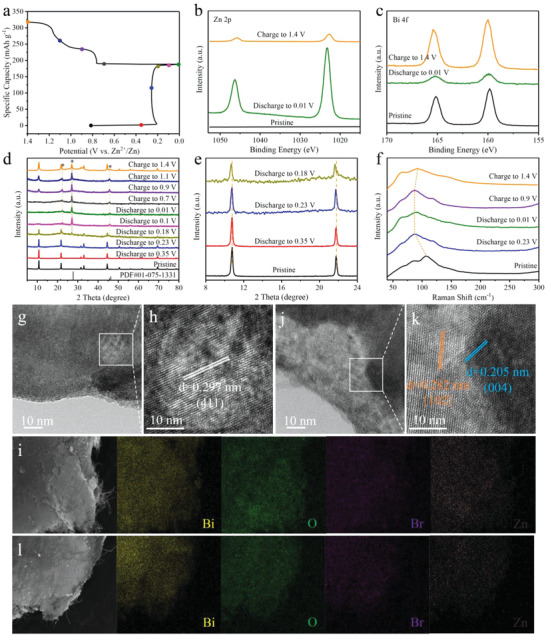
a) Capacity‐voltage curve of UTBiOBr. Ex situ b) Zn 2p XPS spectra, c) Bi 4f XPS spectra, d,e) XRD patterns, and f) Raman spectra of UTBiOBr. HRTEM images and elemental mappings of UTBiOBr at fully g–i) discharged and j–l) charged states.

Ex situ XPS, XRD, Raman, and TEM tests are executed to analyze the energy storage mechanism of UTBiOBr. The first capacity‐voltage curve of UTBiOBr is exhibited in **Figure** **4**a. In the Zn 2p XPS spectra, there is no signal in the initial state and the intensity of Zn 2p increases obviously after being discharged to 0.01 V and then decreases when being charged to 1.4 V, proving the insertion and extraction of Zn^2+^ (Figure 4b). The weak Zn 2p peaks at 1.4 V reflect low level of irreversible reaction of UTBiOBr. The intensity of Bi 4f peak reduces obviously after the electrode is discharged to 0.01 V, suggesting that most bismuth ions are dissolved in the electrolyte at this moment (Figure 4c). The enhanced Bi 4f signal at 1.4 V corresponds to the regeneration of UTBiOBr. The atomic ratios of Zn/Bi of UTBiOBr at various charge/discharge states are collected in Table S4, Supporting Information, and its variation trend is consistent with the above analyses. In the XRD patterns, the peaks of UTBiOBr become weak gradually and a new phase marked by asterisk is found with the reduction of voltage (Figure 4d). Remarkably, the peaks of UTBiOBr shift toward low angles from initial state to discharged to 0.18 V (Figure 4e). Only the characteristic peaks of ZnBr_2_ (PDF#01‐075‐1331) are observed at a fully discharged state. This reveals the insertion‐conversion mechanism of UTBiOBr. As the increase of charge voltage, the peaks of UTBiOBr reappear. The weak peaks of ZnBr_2_ still can be found at a fully charged state, also reflecting low level of irreversible reaction. This also explains the reason for capacity fading in 1st cycle. Raman peaks also show a slightly irreversible shift during the discharge and charge process, which is identical to the results of ex situ XRD and XPS tests (Figure 4f). The structural evolution of UTBiOBr is further studied by TEM images. For UTBiOBr at the fully discharged state, the interplanar distance of 0.297 nm is assigned to the (411) plane of ZnBr_2_ and the uniform distribution of Bi, O, Br, and Zn elements can be seen in element mapping (Figure 4g–i). The (102) and (004) facets of BiOBr are observed again at the fully charged state and the residual Zn^2+^ is determined via element mapping (Figure 4j–l). The aforementioned analyses reveal the insertion‐conversion mechanism of UTBiOBr and slight irreversibility at first cycle. In situ EIS spectra at different charge/discharge states are also collected, as shown in Figure S21a,b, Supporting Information. The resistance variation trends of UTBiOBr and Co‐UTBiOBr are the same. The interfacial resistances (*R*_int_) resulting from the poor hydrophilicity of CNTs decrease as decreasing discharge voltage. And the *R*_int_ at 0.18 V is ignorable because CNTs are coated with some hydrophilic groups or substances in the discharge process. Significantly, the value changes of *R*_o_ and *R*_ct_ in UTBiOBr and Co‐UTBiOBr are small in the process of charge and discharge, reflecting the stable electrochemical processes (Figure S21c,d, Supporting Information). The slopes of sloping lines at low frequency of Co‐UTBiOBr are larger than those of UTBiOBr at initial state, after being discharged to 0.01 V, and after being recharged to 1.4 V, indicating a lower Warburg resistance and a faster Zn^2+^ diffusion of Co‐UTBiOBr.

**Figure 5:**
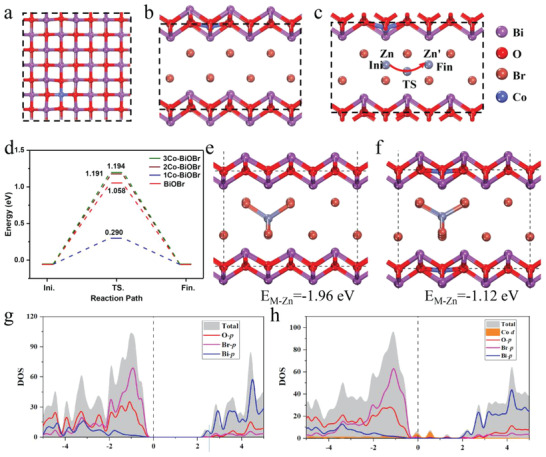
a) Top and b) side views of structures of Co‐doped BiOBr. c) Side view of Zn diffusion in the Co‐doped BiOBr bulk. d) Energy profiles of the corresponding Zn diffusion pathway. Side view of Zn adsorption in the e) BiOBr and f) Co‐doped BiOBr bulks. Calculated DOS of g) BiOBr and h) Co‐doped BiOBr.

The XRD pattern and XPS spectra of Co‐UTBiOBr after 3000 cycles are collected in Figure S22, Supporting Information. The strong characteristic peaks of BiOBr in the XRD pattern and a weak Co 3s XPS peak are still detected, proving its wonderful structural stability and dendrite‐free operation. To reveal the advantages of Co doping, the Zn^2+^ intercalation and diffusion in BiOBr are studied by DFT. It is found that the pristine BiOBr shows a large diffusion barrier of 1.058 eV, indicating a slow Zn^2+^ intercalation rate. The different contents of Co doping are introduced and their top and side views are exhibited in **Figure** **5**a,b, Figures S23 and S24, Supporting Information. To maintain a stable state, one Co is bonded with four O. The Zn^2+^ diffusion barriers of 1Co‐BiOBr, 2Co‐BiOBr, and 3Co‐BiOBr are calculated, as shown in Figure 5c,d. Obviously, a small number of Co doping are beneficial to the Zn^2+^ diffusion and the barrier can be reduced to 0.290 eV. However, excessive Co doping increases diffusion barriers. The adsorption energy (E_M‐Zn_) of 1Co‐BiOBr (−1.12 eV) is smaller than those of BiOBr (−1.96 eV), 2Co‐BiOBr (−2.83 eV), and 3Co‐BiOBr (−3.76 eV), which is one of the key reasons for 1Co‐BiOBr with the lowest diffusion barrier (Figure 5e,f and Figure S25, Supporting Information). The changes in volumes and lattice parameters of BiOBr and 1Co‐BiOBr after Zn^+^ intercalation are calculated, as seen in Figures S26 and S27 and Table S5, Supporting Information. Obviously, the volume change of 1Co‐BiOBr (∆V = 34%) is smaller than that of BiOBr (∆V = 50%), reflecting that the right amount of Co doping results in a better structural stability in the process of Zn^2+^ insertion and extraction. Figure 5g,h shows the projected density of states (DOS) and band structures of BiOBr and 1Co‐BiOBr models. The DOS of 1Co‐BiOBr shows considerable states at the Fermi level, while the states of BiOBr display an obvious bandgap of ≈2.4 eV. This demonstrates the metallic character of 1Co‐BiOBr. DFT calculations explain the reasons for Co‐UTBiOBr with excellent rate ability and long‐term cyclic stability.

**Figure 6:**
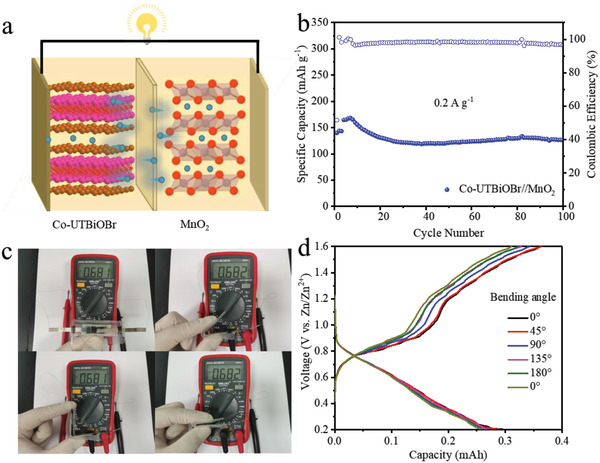
a) Structural illustration of Co‐UTBiOBr//MnO_2_ “rocking chair” ZIBs. b) Cyclic life of Co‐UTBiOBr//MnO_2_ coin cell at 0.2 A g^−1^. c) Digital photos of voltage values of flexible quasi‐solid‐state battery in bending test. d) GCD curves of flexible battery at different bending angles.

The suitable voltage (0.4 V vs Zn^2+^/Zn), considerable specific capacity (150 mAh g^−1^), good rate capability, and long‐term cyclic life of Co‐UTBiOBr in half cell encourage us to further explore its potential as an insertion‐conversion anode in “rocking‐chair” ZIBs. Here, we chose commercial MnO_2_ as cathode because of its high operation potential, high capacity, and low cost. Co‐UTBiOBr//MnO_2_ full cell is assembled and its schematic diagram is exhibited in **Figure** **6**a. There is no need for excessive Zn in this system, dramatically increasing the battery energy density in comparison to conventional Zn metal batteries. The 1st–3rd charge and discharge curves at 0.2 A g^−1^ are displayed in Figure S28, Supporting Information. Its 1st–3rd discharge capacities (coulombic efficiencies) are 168 (62%), 159 (96.3%), and 155 (93.4%) mAh g^−1^, reflecting good compatibility of Co‐UTBiOBr and MnO_2_. Its cyclic life is tested at 0.2 A g^−1^ (Figure 6b). High capacity (126 mAh g^−1^ after 100 cycles) and coulombic efficiencies (≥ 97%) are obtained, reflecting the excellent cycle stability of Co‐UTBiOBr//MnO_2_. Significantly, the specific capacity of Co‐UTBiOBr//MnO_2_ (based on the mass of anode) is superior to the most reported “rocking‐chair” zinc ion batteries such as Na_0.14_TiS_2_//ZnMn_2_O_4_ (105 mAh g^−1^@0.05 A g^−1^),^[^ ^28^ ^]^ h‐WO_3_/3DG//ZnMn_2_O_4_/CB (62 mAh g^−1^@0.1 A g^−1^),^[^ ^24^ ^]^ h‐MoO_3_//Zn_0.2_MnO_2_ (85 mAh g^−1^@0.15 A g^−1^),^[^ ^30^ ^]^ WO_3_/WC//MnO_2_/graphite (118 mAh g^−1^@0.17 A g^−1^),^[^ ^46^ ^]^ Zn_x_MnO_2_//H_2_Ti_3_O_7_ ∙xH_2_O (67 mAh g^−1^@0.2 A g^−1^),^[^ ^31^ ^]^ MoO_2_@NC//Na_3_V_2_(PO_4_)_2_O_2_F (102 mAh g^−1^@0.1 A g^−1^),^[^ ^33^ ^]^ TiSe_2_//VO_2_ (44 mAh g^−1^@0.2 A g^−1^),^[^ ^26^ ^]^ and Zn_2_Mo_6_S_8_//K_0.02_(H_2_O)_0.22_Zn_2.94_[Fe(CN)_6_]_2_ (62 mAh g^−1^@0.064 A g^−1^).^[^ ^47^ ^]^ Co‐UTBiOBr//MnO_2_ quasi‐solid‐state flexible battery is also designed. In the bending test, the stable voltage value of the flexible battery is observed, reflecting its good structural stability (Figure 6c). GCD curves of flexible batteries are collected at different bending states from 0° to 180°. The similar shape of GCD curves and the negligible capacity decay prove the successful design of a quasi‐solid‐state flexible battery. The excellent battery performance of the Co‐UTBiOBr//MnO_2_ “rocking chair” battery further reflects the great potential application of Co‐UTBiOBr (Figure 6d).

## 3 Conclusions

In summary, a few‐atomic‐layered Co‐doped BiOBr has been successfully designed through an one‐step hydrothermal method. A free‐standing flexible Co‐UTBiOBr@CNTs electrode is prepared via a simple vacuum filtration. Decreased nanosheet thickness and Co‐doping are beneficial to fast Zn^2+^ transport and repressed volume variation, and CNTs and Co‐doping favor electron transfer, which is proved by experimental and theoretical points. Furthermore, the insertion‐conversion mechanism of UTBiOBr is revealed by ex situ XPS, XRD, Raman, and TEM tests. Significantly, Co‐UTBiOBr displays satisfactory rate ability and cycling performance at different mass loading of 5, 10, and 15 mg cm^−2^. And Co‐UTBiOBr//MnO_2_ “rocking chair” ZIB exhibits good electrochemical performances during cyclic test and their flexible battery shows excellent stability. This work gives a reference for the design of high‐performance anode with ultrahigh mass loading.

## 4 Experimental Section

### Preparation of Co‐Doped BiOBr Ultrathin Nanosheets

First, 1 mmol Bi(NO_3_)_3_∙5H_2_O (98%, Macklin), 1 mmol ionic liquid [C_16_mim]Br (1‐hexadecyl‐3‐methylimidazolium bromide) (99%, Aladdin), and a certain amount (0.04, 0.06, and 0.08 mmol) of Co(NO_3_)_2_ 6H_2_O (99%, Aladdin) were dissolved into 20 mL H_2_O. The pH of the solution was adjusted to 1 via HNO_3_ (65%–68%, Xilong scientific) and the solution was stirred for 30 min. Last, the above solution was transferred into a PTFE‐lined stainless steel autoclave and heated in an oven at 140 °C for 24 h. The products were centrifuged, washed, and dried to obtain Co‐doped BiOBr ultrathin nanosheets. The products using 0.04, 0.06, and 0.08 mmol Co(NO_3_)_2_ 6H_2_O were denoted as 0.04Co‐UTBiOBr, 0.06Co‐UTBiOBr (Co‐UTBiOBr), and 0.08Co‐UTBiOBr. UTBiOBr was synthesized via the same hydrothermal method but without the addition of Co(NO_3_)_2_∙5H_2_O.

### Preparation of Thick BiOBr Nanosheets

1 mmol Bi(NO_3_)_3_∙5H_2_O and 1 mmol KBr (99%, aladdin) were dissolved into 20 mL H_2_O. The suspension was stirred for 60 min at room temperature. The product was centrifuged, washed, and dried to obtain thick BiOBr.

### Preparation of BiOBr‐Based Flexible Electrodes

To prepare BiOBr‐based flexible electrodes, thick BiOBr/UTBiOBr/Co‐UTBiOBr and multi‐walled CNTs (purity: >95%, diameter: 8–15 nm, length: ≈50 nm, XFNANO) with a weight ratio of 8 : 2 were dispersed in ethanol. Then the solution was filtrated with a microporous filtration membrane. The obtained films were dried in a vacuum oven to form a free‐standing and flexible films. It was cut into small disks for an electrochemical performance test. The areal weight of the flexible electrode was 5 mg cm^−2^. Similarly, flexible Co‐UTBiOBr electrodes with high mass loading of 10 and 15 mg cm^−2^ were prepared.

### Characterization

The crystal structure, molecular structure, and elemental composition were analyzed through XRD (D‐MAX 2200 VPC, Rigaku) pattern with Cu K*α* radiation (*λ* = 1.5418 A), Raman spectra (inVia, Renishaw) with a laser length of 514 nm, FT‐IR (Nicolet 6700, Thermo Scientific) spectra, and X‐ray photoelectron spectra (XPS, ESCALab250, Thermo VG) corrected by C 1s line at 284.6 eV. The SEM (Gemini500, Zeiss), AFM (SPM‐9500 J3, Bruker), and TEM (Tecnai G2 F30, FEI) were utilized to observe the morphology evolution of materials. Nitrogen adsorption/desorption isotherms were recorded to count the specific surface area and pore volume of materials (ASAP2460, Micromeritics).

### Electrochemical Measurements

The cell tests were performed in a 2025‐type coin cell. For half‐cell, BiOBr‐based nanopapers and Zn foil were used as working electrodes and counter/reference electrodes respectively. The commercial MnO_2_ powder was pasted on stainless steel foil via preparing slurry with 70% active material, 20% acetylene black, and 10% poly(vinylidene fluoride). The electrolyte was aqueous solution with 2 mol L^−1^ Zn(CF_3_SO_3_)_2_. Glass fiber membrane (Whatman, GF/D) worked as a separator. For quasi‐solid‐state battery, cathode and anode were commercial MnO_2_ and Co‐UTBiOBr and their mass ratio was about 1:1. To prepare a solid electrolyte separator with good flexibility, 1.5 g gelatin was dissolved into 6 mL 1 mol L^−1^ ZnSO_4_ aqueous solution and stirred for 30 min at 60 °C. Then a suitable size of glass fiber membrane was immersed in the above solution for 5 min, taken out, and left for 2 h at room temperature before it was used as separator. The flexible quasi‐solid‐state battery was encapsulated via using PA/PE film as outer packing. The battery tests were performed in the voltage window of 0.01–1.4 (half cell) and 0.2–1.6 V (“rocking chair” battery) by a NEWARE battery test system (CT‐4008‐5V20mA‐164, Shenzhen, China). CV curves and EIS (Frequency: 0.1–100 000 Hz) were tested by utilizing an electrochemical workstation (DH7000, Jiangsu Donghua Analysis Instruments Co. Ltd.). The GITT tests were recorded via a NEWARE battery test system with a current pulse of 30 mA g^−1^ and a potential relaxation step for 2 h at open‐circuit voltage.

### Details of Calculation

The dominant capacitive behavior or diffusion process can be determined according to equation: *i* = a*ν* ^*b*^, where *i* and *v* present peak current and scan rate. The capacitive behavior or diffusion process will dominate when *b* was about 1 or 0.5.^[^ ^12^, ^48^ ^]^ The accurate pseudocapacitive and diffusion‐controlled contributions of electrodes can be measured according to equation: *i* (V) = *k*_1_ *ν* + *k*_2_ *ν* ^1/2^, where *k*_1_ *v* and *k*_2_ *v* ^1/2^ match with capacitance and intercalation parts, *ν* is the scan rate.^[^ ^10^, ^49^ ^]^ The Zn^2+^ diffusion coefficients of electrodes were counted by GITT curves and Fick's second law: $D\;=\frac{4}{\pi \tau }\;{\left(\frac{m_{B}V_{M}}{M_{B}S}\right)}^{2}{\left(\frac{\Delta E_{S}}{\Delta E_{\tau }}\right)}^{2}$, where *m*_B_, *S*, *V*_M_, and *M*_B_ represent weight, surface area, the molar volume, and the molar mass of active material, *τ* corresponds to the time of current pulse, ∆*E*_S_ represents the steady‐state voltage change by the current pulse, and ∆*E_*τ*_* is the potential difference of constant current pulse charging or discharging.^[^ ^50^ ^]^

### DFT Calculations

DFT calculations were performed by utilizing the Dmol^3^ program with the generalized gradient approximation (GGA) in the form of the Perdew–Burke–Ernzerhof (PBE) exchange‐correlation functional, as implemented in the Materials Studio package. The real‐space global cutoff radius was set to be 4.0 Å. The structure optimization and transition‐state search were achieved with a 2×2×3 k‐point grid. The double numerical (DND) basis set and semicore pseudopotential were utilized to process atomic orbitals and core electrons respectively. The reaction pathways for the Zn diffusion in the 3×3×1 BiOBr and xCo‐BiOBr (x = 1, 2, 3) bulks were figured via using a combination of linear and quadratic synchronous transit (LST/QST) method. The convergence criteria for geometrical optimization and DOS calculation were 1.0×10^−5^ Hartree for energy change, 4.0×10^−3^ Hartree for gradient, and 5.0×10^−3^ Hartree for displacement.

## Conflict of Interest

The authors declare no conflict of interest.

## Supporting information

Supporting Information

## Data Availability

The data that support the findings of this study are available from the corresponding author upon reasonable request.
