# Supplementary material for: Few‐Atomic‐Layered Co‐Doped BiOBr Nanosheet: Free‐Standing Anode with Ultrahigh Mass Loading for “Rocking Chair” Zinc‐Ion Battery
Source: Adv Sci (Weinh). 2022 Sep 13;9(32):2204087. doi: 10.1002/advs.202204087 (PMC9661821; doi:10.1002/advs.202204087)
Supplement: Supplementary file 1 — Supporting Information [file ADVS-9-2204087-s001.pdf]

ADVANCED SCIENCE
Open Access

Supporting Information

for *Adv. Sci.*, DOI 10.1002/advs.202204087

Few-Atomic-Layered Co-Doped BiOBr Nanosheet: Free-Standing Anode with Ultrahigh Mass Loading for “Rocking Chair” Zinc-Ion Battery

Bei Long, Qing Zhang, Tengfei Duan, Ting Song, Yong Pei, Xianyou Wang, Chunyi Zhi, Xiongwei Wu, Qianyu Zhang and Yuping Wu*

Supporting Information

Few-atomic-layered Co-doped BiOBr nanosheet: free-standing anode with ultrahigh mass loading for "rocking chair" zinc-ion battery

Bei Long, Qing Zhang, Tengfei Duan, Ting Song, Yong Pei, Xianyou Wang, Chunyi Zhi, Xiongwei Wu, Qianyu Zhang,* Yuping Wu*

B. Long, Q. Zhang, T. Duan, T. Song, Prof. Y. Pei, Prof. X. Wang
School of Chemistry
Xiangtan University, Xiangtan, 411105, P. R. China
Prof. X. Wu
School of Chemistry and Materials Science
Hunan Agricultural University, Changsha, 410128, P. R. China
E-mail: wxw@hunau.edu.cn (X. Wu)
Prof. Q. Zhang
College of Materials Science and Engineering
Sichuan University, Chengdu, Sichuan, 610064
E-mail: zhangqianyu@scu.edu.cn (Q. Zhang)
Prof. Y. Wu
School of Energy and Environment
Southeast University, Nanjing, 211189, P. R. China
Prof. C. Zhi
Department of Materials Science and Engineering
City University of Hong Kong, Hong Kong, 999077, P. R. China

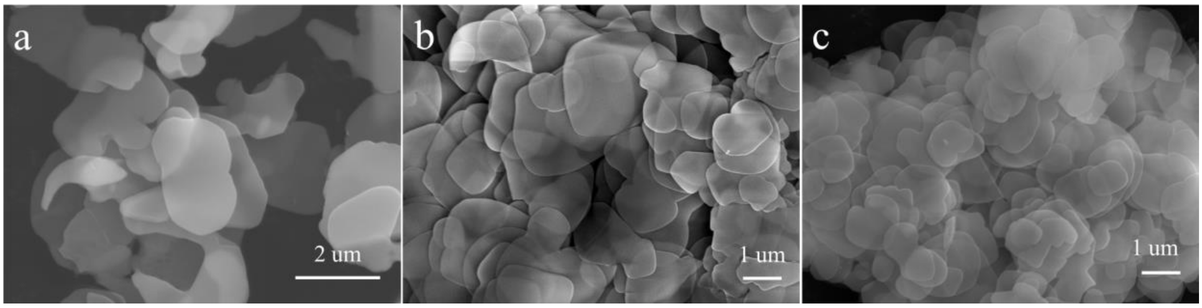

Figure S1. SEM images of a) thick BiOBr, b) UTBiOBr and c) Co-UTBiOBr.

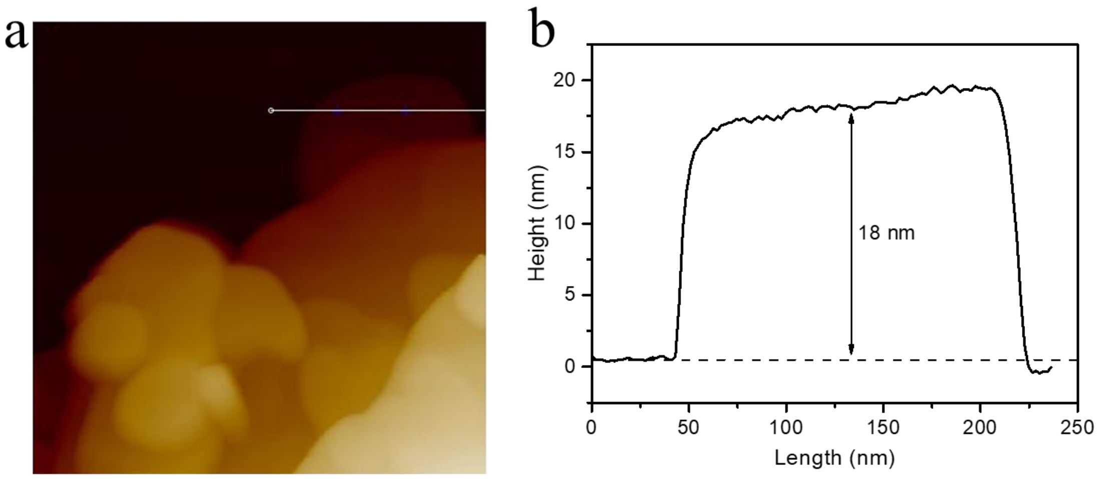

Figure S2. a) AFM image and b) corresponding height profile of thick BiOBr.

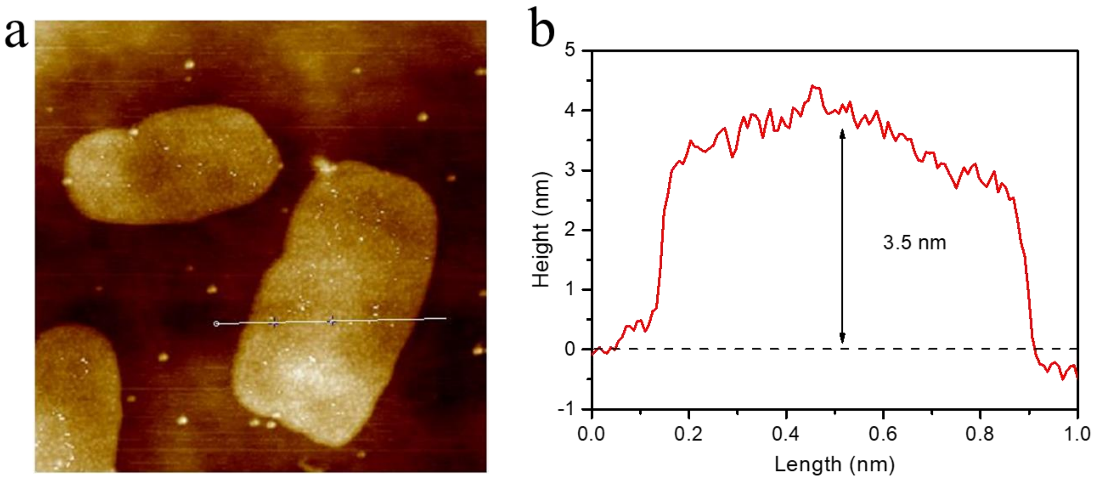

Figure S3. a) AFM image and b) corresponding height profile of UTBiOBr.

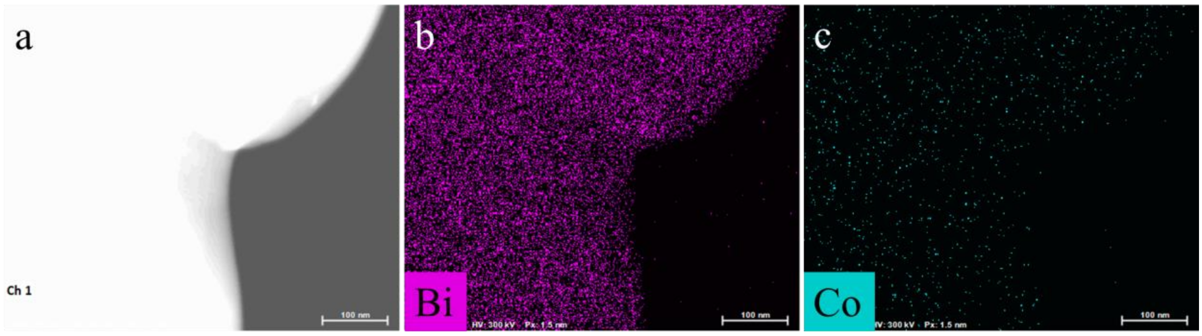

Figure S4. Elemental mapping of Co-UTBiOBr.

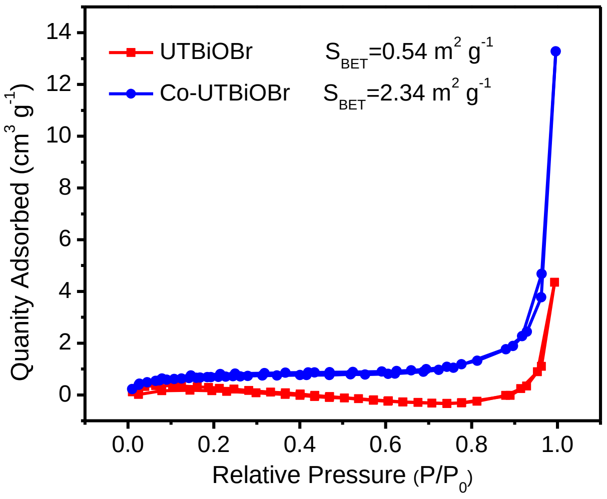

Figure S5. Nitrogen adsorption/desorption isotherms of UTBiOBr and Co-UTBiOBr.

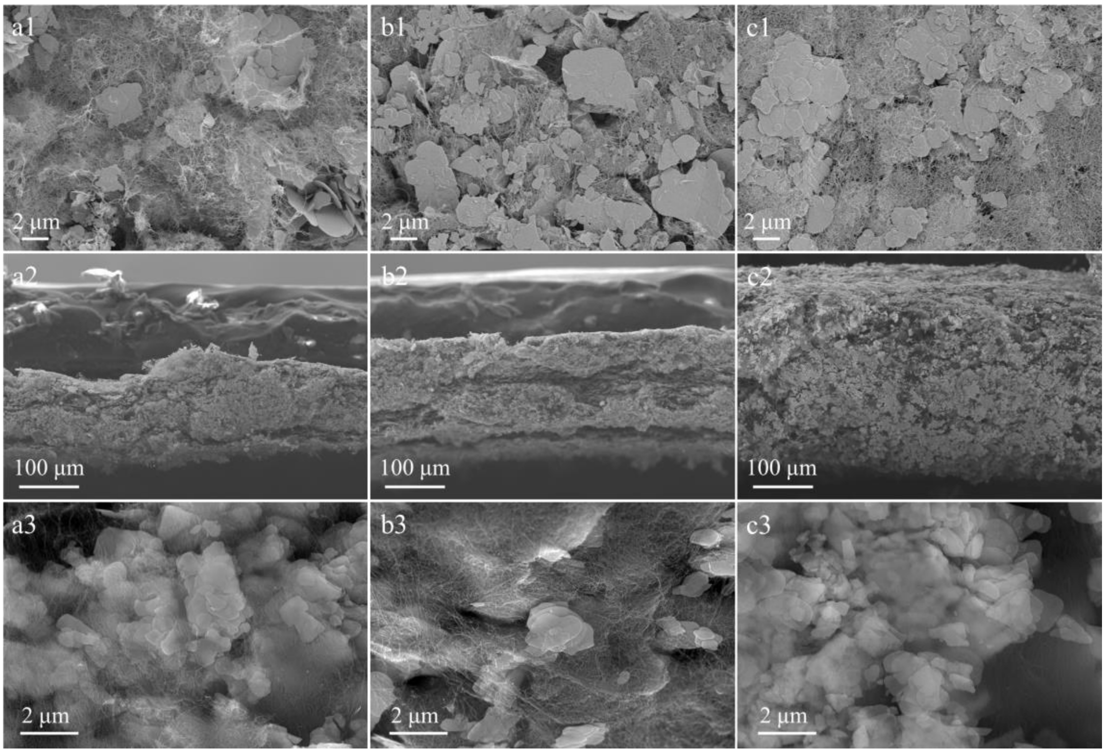

Figure S6. SEM images of top-view (x1) and cross-section (x2, x3) of free-standing Co-UTBiOBr electrodes with mass loading of a1-a3) 5, b1-b3) 10 and c1-c3) 15 mg cm^{-2}.

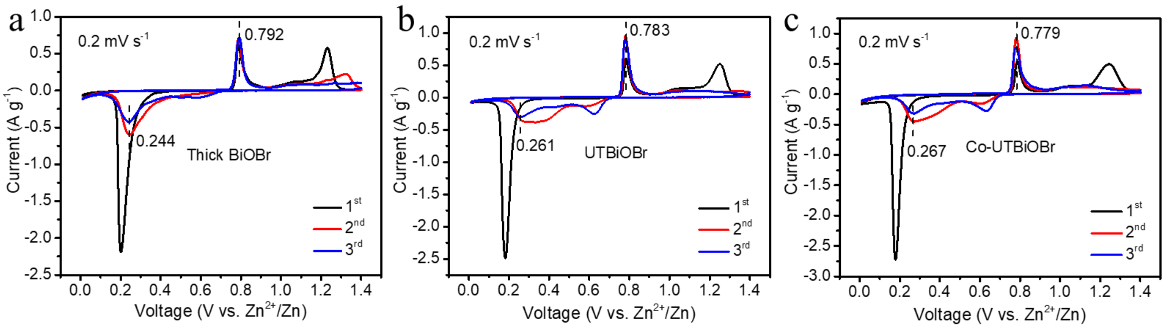

Figure S7. CV curves of a) thick BiOBr, b) UTBiOBr and (c) Co-UTBiOBr with mass loading of 5 mg cm^{-2}.

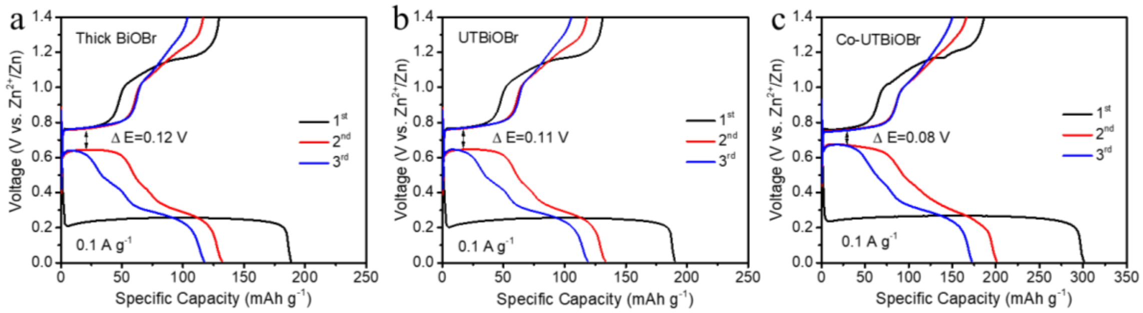

Figure S8. GCD curves of a) thick BiOBr, b) UTBiOBr and c) Co-UTBiOBr with mass loading of 5 mg cm^{-2}.

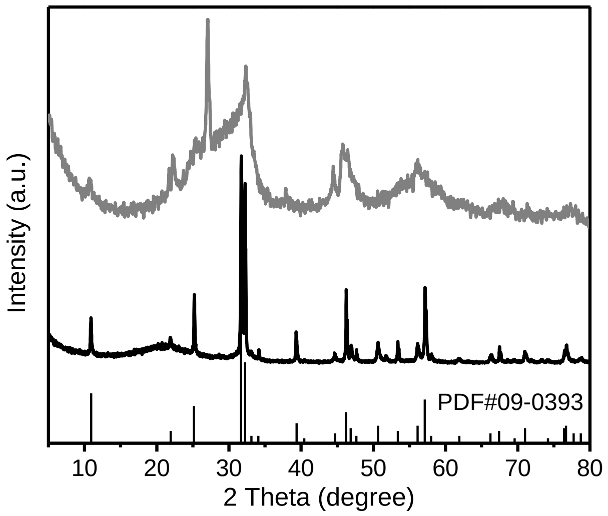

Figure S9. XRD patterns of thick BiOBr before cycle (black) and after 1 cycle (gray).

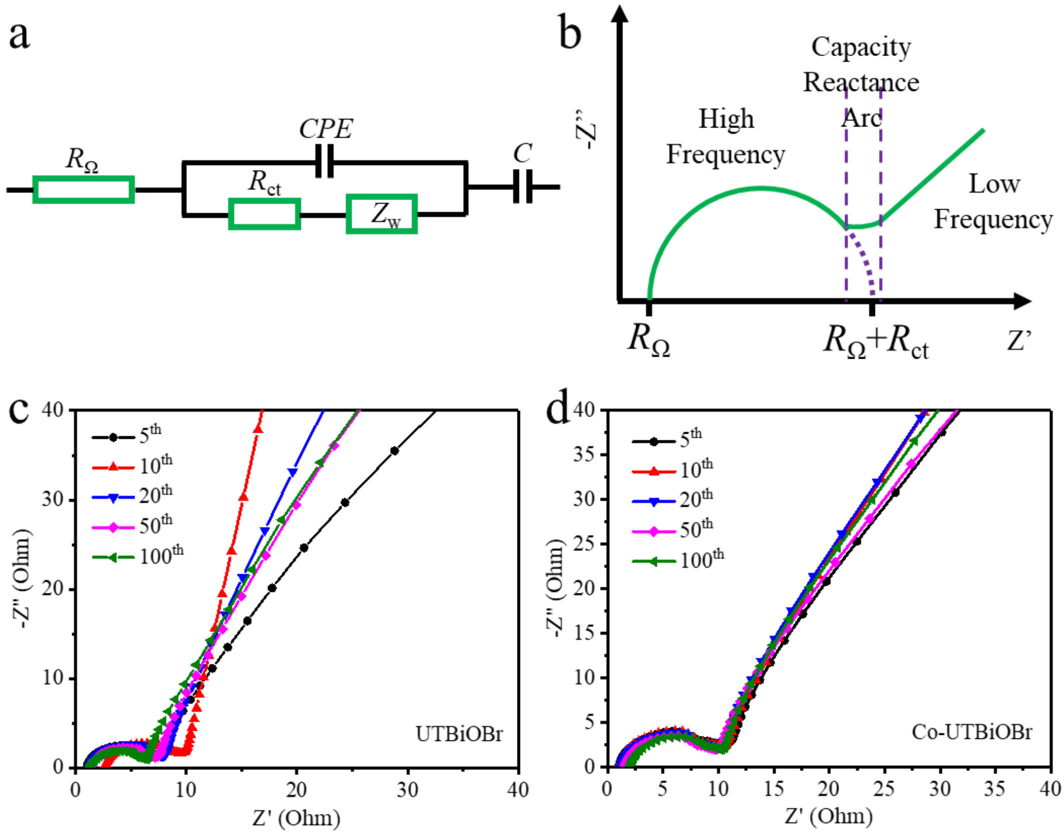

Figure S10. a) Modeled equivalent circuit of EIS. b) Schematic of EIS, where R_{Ω} stands for the electrolyte resistance, R_{ct} the charge transfer resistance, Z_w the "Warburg"-type element related to Zn ion diffusion, *CPE* the constant phase element and *C* the potential-dependent capacitance. In situ EIS of c) UTBiOBr and d) Co-UTBiOBr with mass loading of 5 mg cm^{-2}.

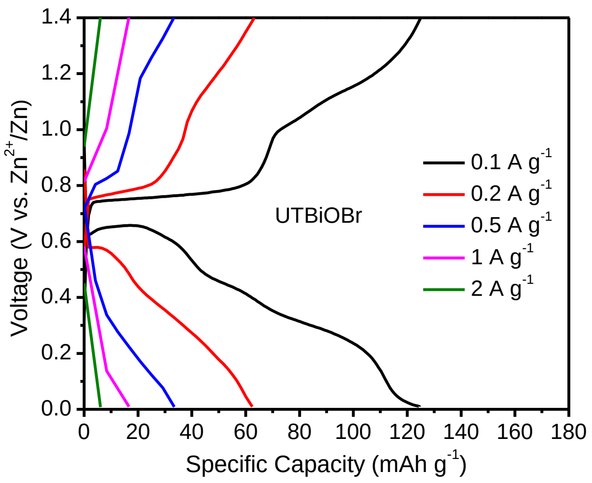

Figure S11. GCD curves at different current densities of UTBiOBr with mass loading of 5 mg cm^{-2}.

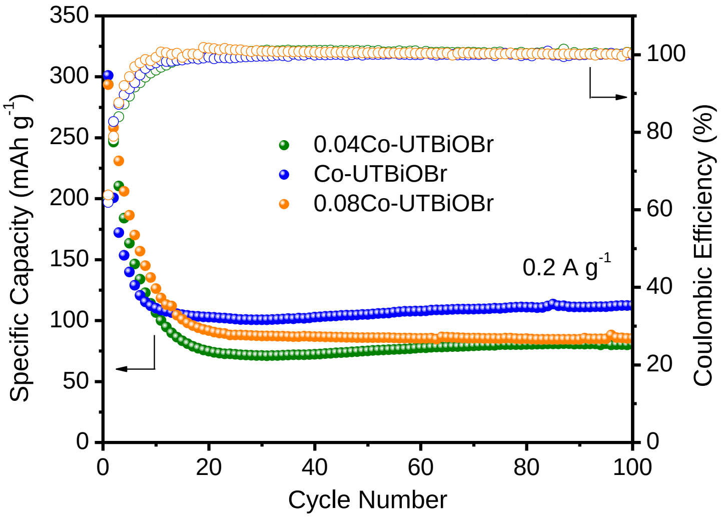

Figure S12. Cyclic life of 0.04Co-UTBiOBr, Co-UTBiOBr (0.06Co-UTBiOBr) and 0.08Co-UTBiOBr with mass loading of 5 mg cm^{-2}.

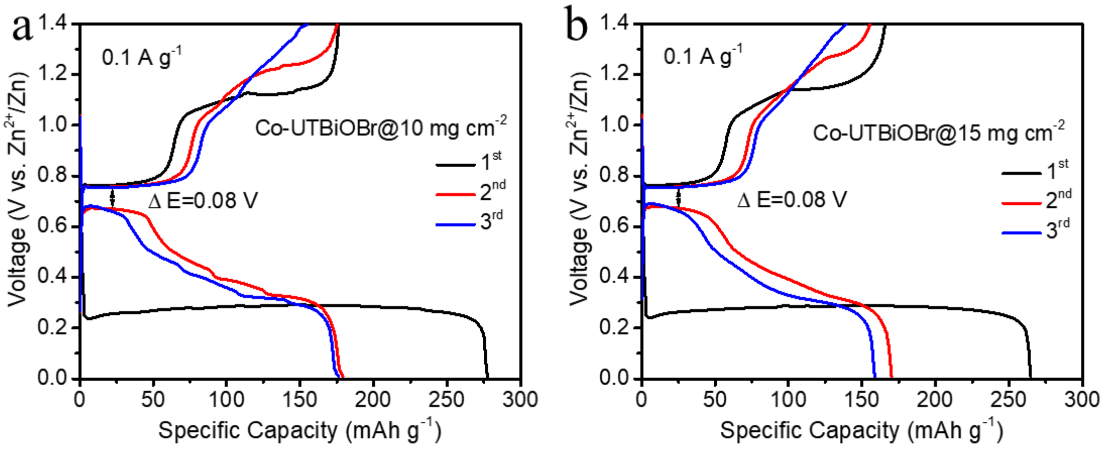

Figure S13. 1st-3rd GCD curves of Co-UTBiOBr with mass loading of a) 10 and b) 15 mg cm^{-2}.

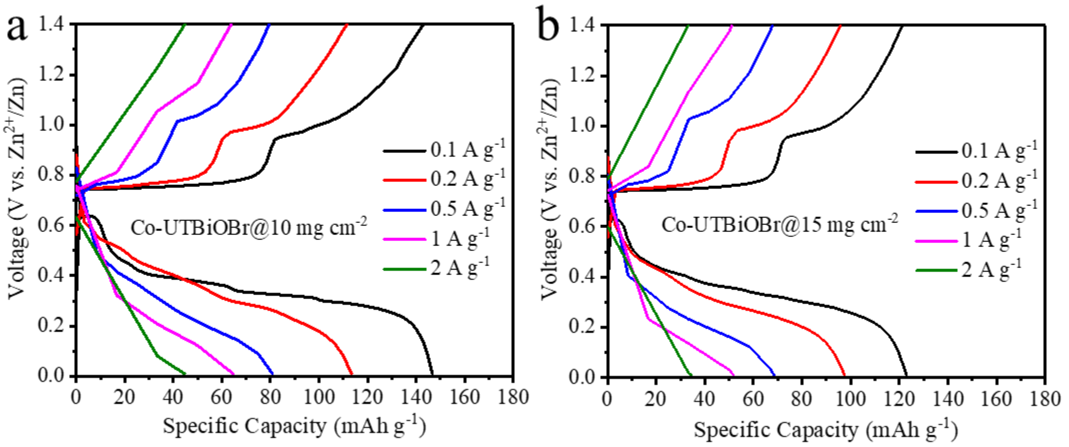

Figure S14. GCD curves of Co-UTBiOBr with mass loading of a) 10 and b) 15 mg cm^{-2}.

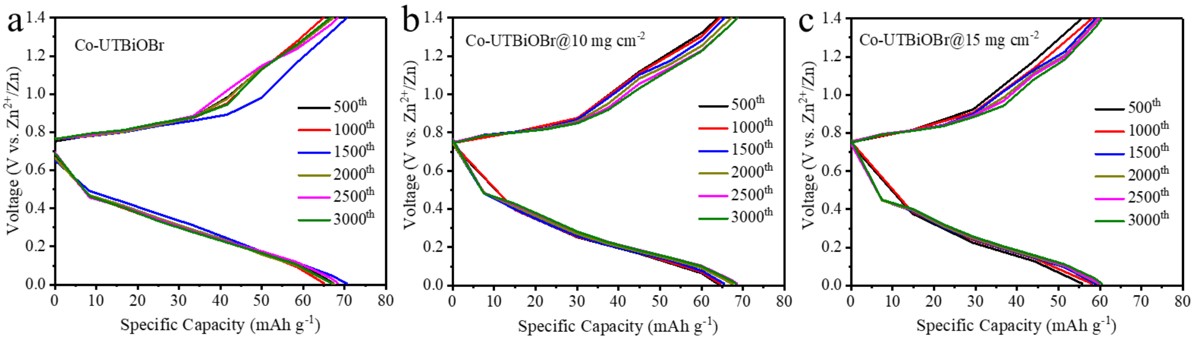

Figure S15. GCD curves of Co-UTBiOBr with mass loading of a) 5, b) 10 and c) 15 mg cm^{-2} during cyclic tests.

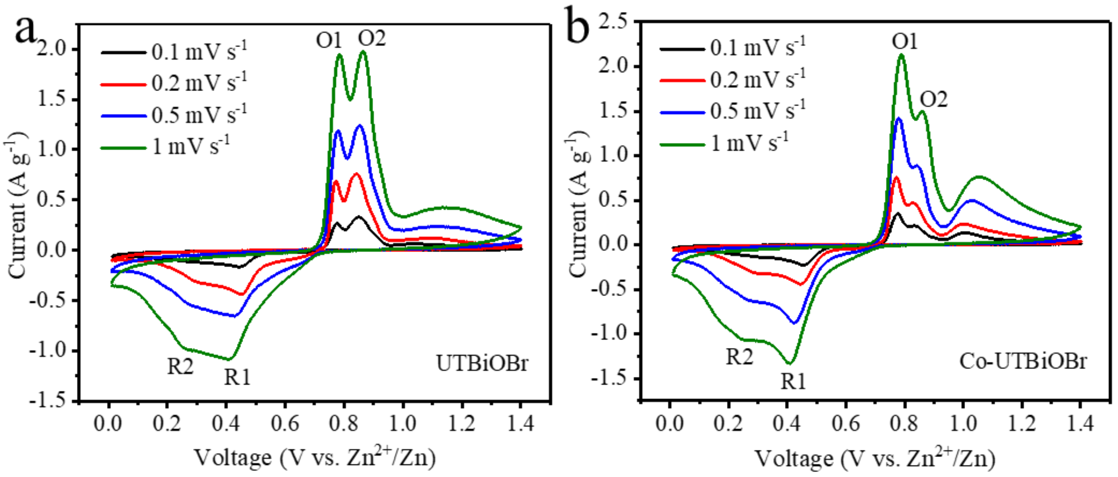

Figure S16. CV curves from 0.1 to 1 mV s^{-1} of a) UTBiOBr and b) Co-UTBiOBr with mass loading of 5 mg cm^{-2}..

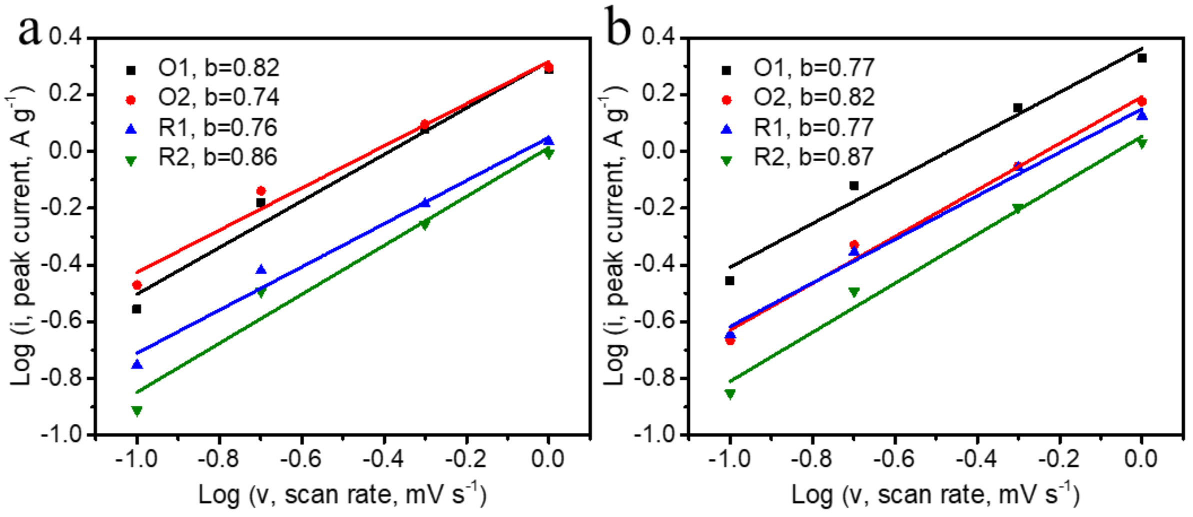

Figure S17. Log(i) versus log(v) plots at specific peak currents in CV curves of a) UTBiOBr and b) Co-UTBiOBr.

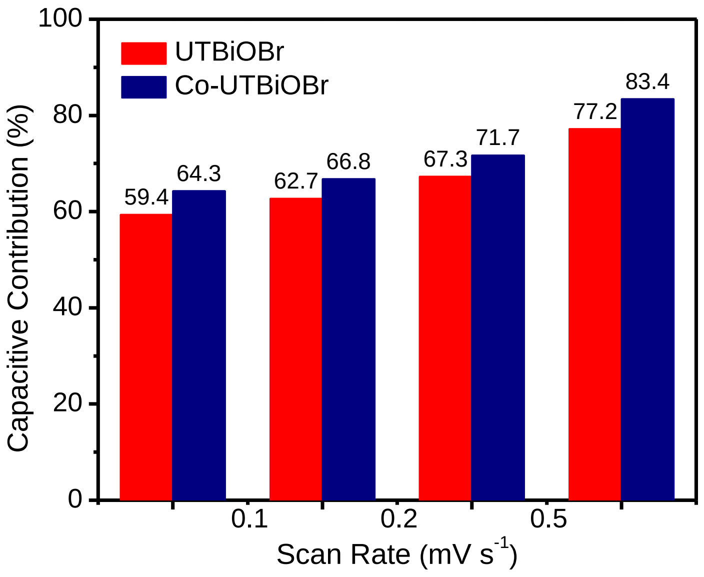

Figure S18. Pseudocapacitive contributions at different scan rates of UTBiOBr and Co-UTBiOBr.

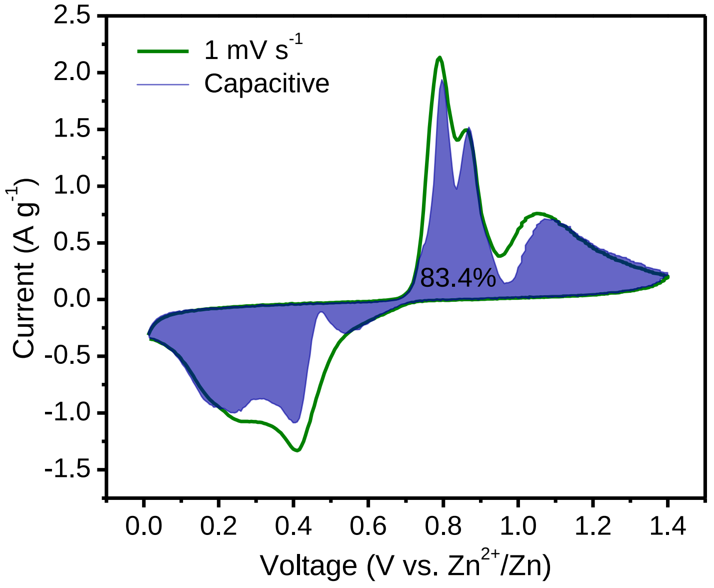

Figure S19. CV curve with the calculated pseudocapacitive fraction shown by the shaded area at 1 mV s^{-1} of Co-UTBiOBr.

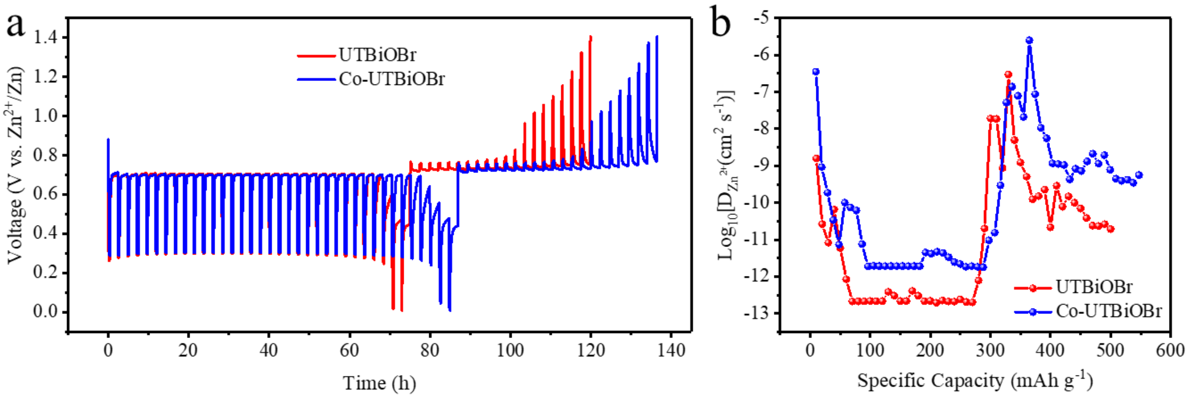

Figure S20. a) Charge-discharge curves in the GITT measurements and b) corresponding Zn^{2+} diffusion coefficients at different discharge/charge states of UTBiOBr and Co-UTBiOBr.

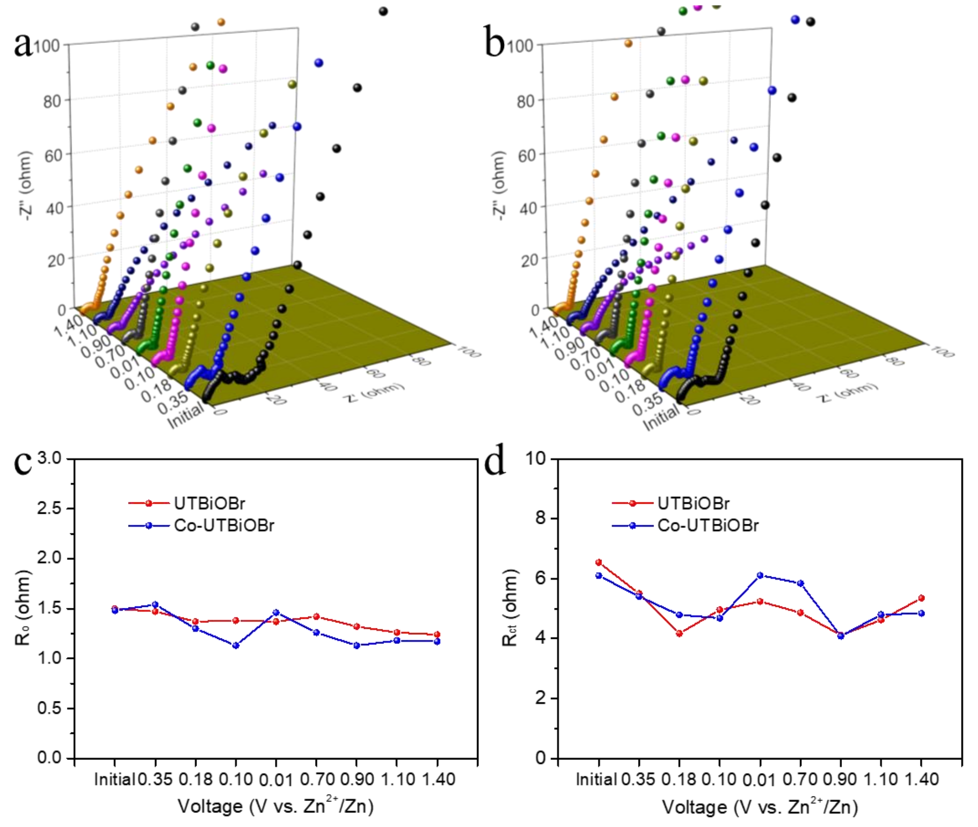

Figure R21. In situ EIS spectra of a) UTBiOBr and b) Co-UTBiOBr. c) R_o and d) R_{ct} of UTBiOBr and Co-UTBiOBr at different charge and discharge states.

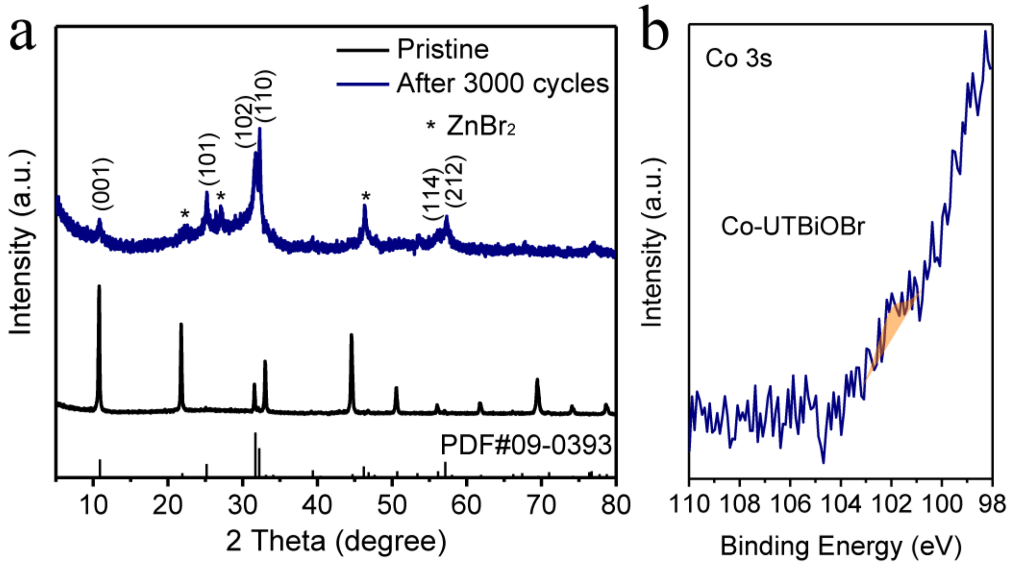

Figure S22. a) XRD pattern and b) Co 3s XPS spectrum of Co-UTBiOBr after 3000 cycles.

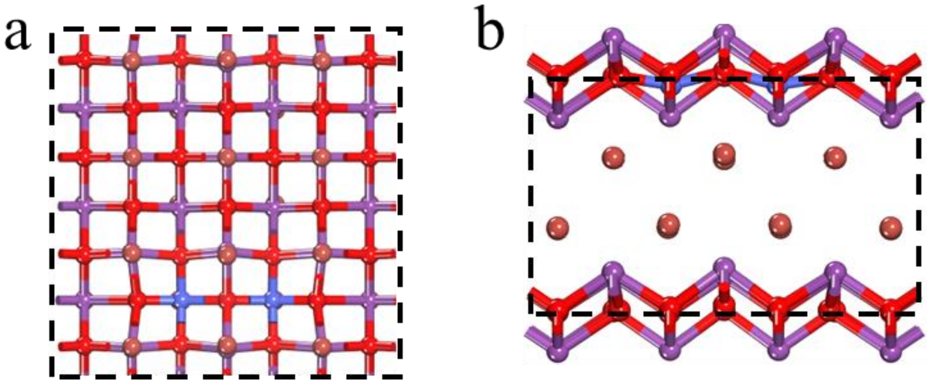

Figure S23. a) Top and b) side views of the 2Co-BiOBr bulk.

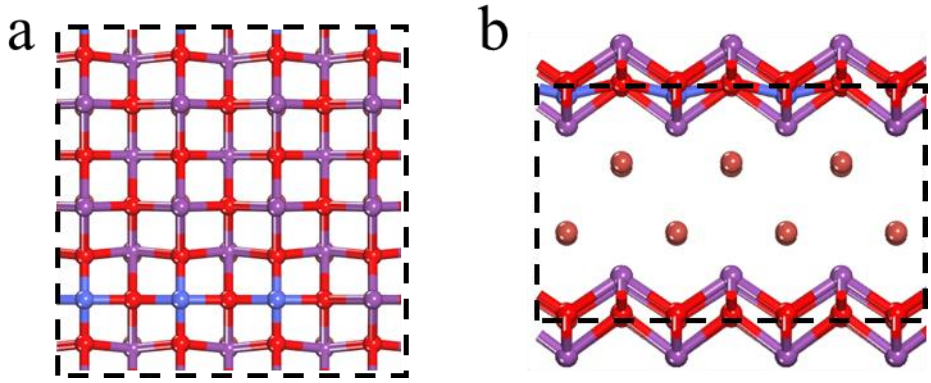

Figure S24. a) Top and b) side views of the 3Co-BiOBr bulk.

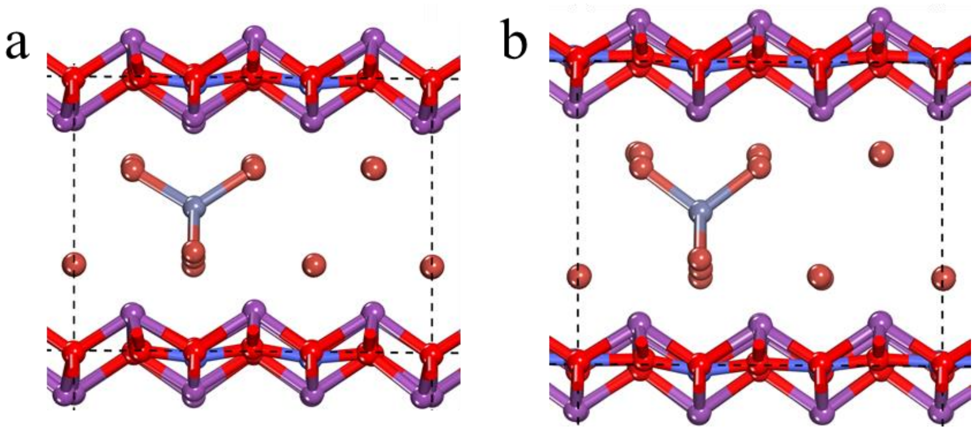

Figure S25. Side views of (a) 2Co-BiOBr@Zn and (b) 3Co-BiOBr@Zn bulks.

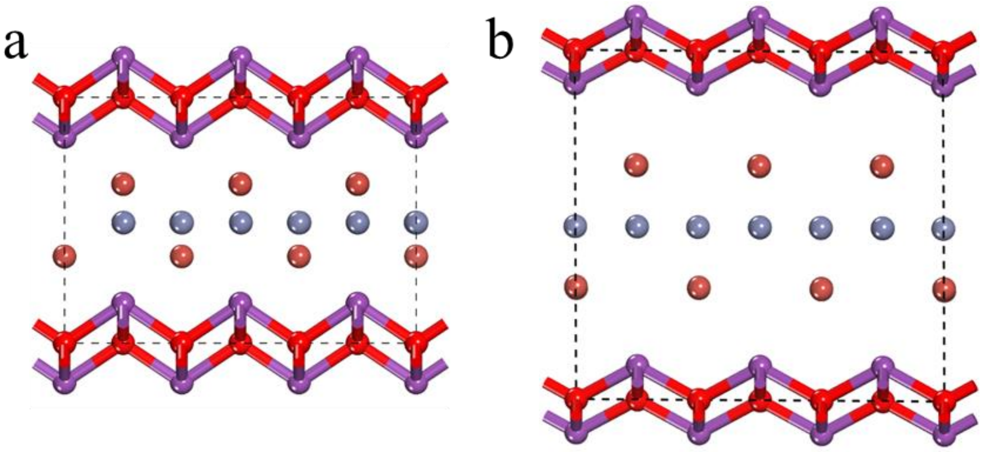

Figure S26. Side views of BiOBr@18Zn a) at initial state and b) after relaxation.

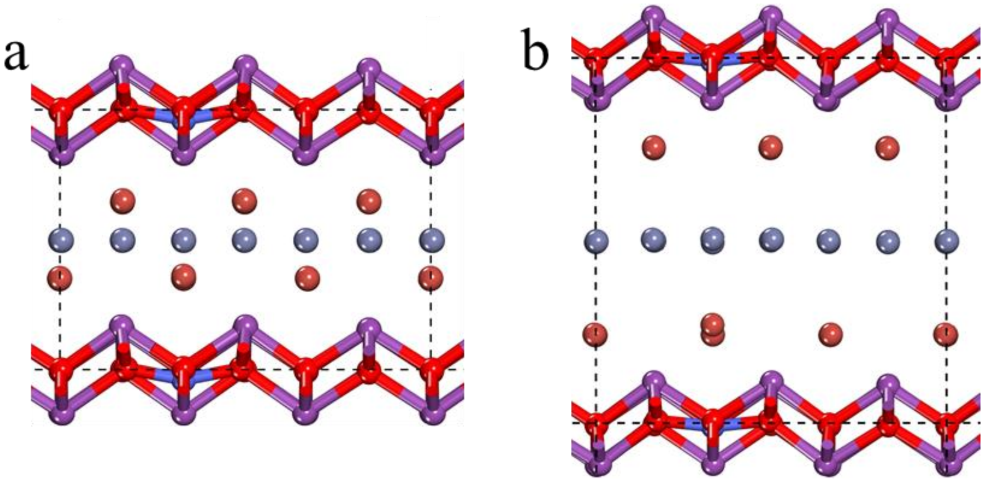

Figure S27. Side views of Co-BiOBr@18Zn a) at initial state and b) after relaxation.

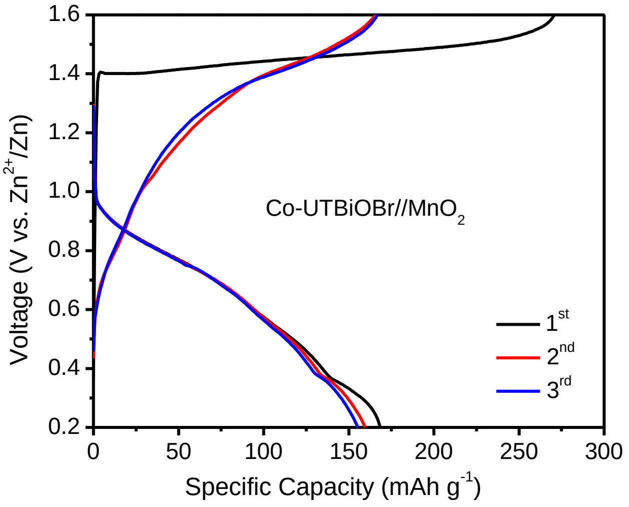

Figure S28. The 1st-3rd GCD curves of Co-UTBiOBr//MnO_2

Table S1. Omic resistances (R_o), charge transfer resistances (R_{ct}) and slopes related to Warburg resistances of UTBiOBr during cyclic tests.

UTBiOBr	R_o	R_{ct}	Slope
**After 5 cycles**	1.440	9.635	1.064
**After 10 cycles**	2.573	10.522	4.237
**After 20 cycles**	1.253	8.519	1.871
**After 50 cycles**	1.384	7.646	1.469
**After 100 cycles**	1.281	7.025	1.591

Table S2. Omic resistances (R_o), charge transfer resistances (R_{ct}) and slopes related to Warburg resistances of Co-UTBiOBr during cyclic tests.

Co-UTBiOBr	R_o	R_{ct}	Slope
**After 5 cycles**	0.988	10.808	1.863
**After 10 cycles**	1.018	11.106	1.998
**After 20 cycles**	1.211	10.545	1.980
**After 50 cycles**	1.484	10.738	1.858
**After 100 cycles**	1.816	10.838	1.959

Table S3. A comparison of electrochemical performances of reported anodes in ZIBs.

Anode	Specific capacity	Cycle stability	Mass loading
Co-UTBiOBr	150/140/130 mAh g^{-1}@0.1 A g^{-1}	≈100%@1 A g^{-1} @3000 cycles	5/10/15 mg cm^{-2}
**CuS@CTMAB[1]**	367 mAh g^{-1} @0.1 A g^{-1}	61%@2 A g^{-1} @3000 cycles	1 mg cm^{-2}
**CuS@CTAB[2]**	225 mAh g^{-1} @0.1 A g^{-1}	87%@10 A g^{-1} @3400 cycles	1 mg cm^{-2}
**$Na_{0.14}TiS_2$[3]**	140 mAh g^{-1} @0.05 A g^{-1}	77%@0.5 A g^{-1} @5000 cycles	5-6 mg cm^{-2}
**$Cu_{2-x}Te$[4]**	158 mAh g^{-1} @0.03 A g^{-1}	≈100%@0.6 A g^{-1} @2000 cycles	2-5 mg cm^{-2}
**MoO_2@NC[5]**	150 mAh g^{-1} @0.1 A g^{-1}	61%@5 A g^{-1} @3000 cycles	1-1.5 mg cm^{-2}
**NHVO@$Ti_3C_2T_x$[6]**	514 mAh g^{-1} @0.1 A g^{-1}	84%@5 A g^{-1} @6000 cycles	1-2 mg cm^{-2}
**$H_2Ti_3O_7\cdot xH_2O$[7]**	100 mAh g^{-1} @0.2 A g^{-1}	87%@0.2 A g^{-1} @1400 cycles	4 mg cm^{-2}
**WO_3/WC[8]**	164 mAh g^{-1} @0.1 A g^{-1}	90%@1 A g^{-1} @1000 cycles	1.4 mg cm^{-2}
**NI[9]**	166 mAh g^{-1} @0.1 A g^{-1}	99%@3 A g^{-1} @2000 cycles	/
**H-MoO_3[10]**	120 mAh g^{-1} @0.2 A g^{-1}	≈100%@0.3 A g^{-1} @100 cycles	0.8 mg cm^{-2}
**AQ[11]**	200 mAh g^{-1} @0.2 A g^{-1}	84%@0.2 A g^{-1} @200 cycles	2-5 mg cm^{-2}
**$TiSe_2$[12]**	128 mAh g^{-1} @0.2 A g^{-1}	70%@0.8 A g^{-1} @300 cycles	/

Table S4. The atomic ratios of Zn/Bi of UTBiOBr at various charge/discharge states.

Initial	Discharge to 0.23 V	Discharge to 0.01 V	Charge to 0.9 V	Charge to 1.4 V
0 : 1	0.71 : 0.29	0.96 : 0.04	0.82 : 0.18	0.41 : 0.59

Table S5. The initial volume, volume after relaxation, volume change and lattice parameter of BiOBr-based systems.

System	V_0(Å^3)	V_1(Å^3)	ΔV(%)	a(Å)	b(Å)	c(Å)
**BiOBr**	**-**	**-**	**-**	**12.00**	**12.00**	**8.65**
**BiOBr@18Zn**	**1246.5**	**1867.4**	**49.80737**	**12.68**	**12.71**	**11.79**
**1Co-BiOBr**	**-**	**-**	**-**	**11.93**	**11.93**	**8.85**
**1CoBiOBr@18Zn**	**1260.4**	**1691.8**	**34.2234**	**11.75**	**11.78**	**12.23**

Reference

[1] Z. Lv, B. Wang, M. Ye, Y. Zhang, Y. Yang, C. C. Li, *ACS Appl. Mater. Interfaces* **2022**, *14*, 1126.

[2] J. Zhang, Q. Lei, Z. Ren, X. Zhu, J. Li, Z. Li, S. Liu, Y. Ding, Z. Jiang, J. Li, Y. Huang, X. Li, X. Zhou, Y. Wang, D. Zhu, M. Zeng, L. Fu, *ACS Nano* **2021**, *15*, 17748.

[3] W. Li, K. Wang, S. Cheng, K. Jiang, *Adv. Energy Mater.* **2019**, *9*, 1900993.

[4] W. Li, Y. Ma, P. Li, X. Jing, K. Jiang, D. Wang, *Adv. Energy Mater.* **2021**, *11*, 2102607.

[5] B. Wang, J. Yan, Y. Zhang, M. Ye, Y. Yang, C. C. Li, *Adv. Funct. Mater.* **2021**, *31*, 2102827.

[6] X. Wang, Y. Wang, Y. Jiang, X. Li, Y. Liu, H. Xiao, Y. Ma, Y. Y. Huang, G. Yuan, *Adv. Funct. Mater.* **2021**, *31*, 2103210.

[7] Y. Liu, X. Zhou, X. Wang, G. Chen, R. Liu, Y. Ma, Y. Bai, G. Yuan, *Chem. Eng. J.* **2021**, *420*, 129629.

[8] J. Cao, D. Zhang, Y. Yue, X. Wang, A. Srikhaow, C. Sriprachuabwong, A. Tuantranont, X. Zhang, Z.-S. Wu, J. Qin, *Chem. Eng. J.* **2021**, *426*, 131893.

[9] Y. Liu, M. Huang, F. Xiong, J. Zhu, Q. An, *Chem. Eng. J.* **2022**, *428*, 131092.

[10] T. Xiong, Y. Zhang, Y. Wang, W. S. V. Lee, J. Xue, *J. Mater. Chem. A* **2020**, *8*, 9006.

[11] L. Yan, X. Zeng, Z. Li, X. Meng, D. Wei, T. Liu, M. Ling, Z. Lin, C. Liang, *Mater. Today Energy* **2019**, *13*, 323.

[12] L. Wen, Y. Wu, S. Wang, J. Shi, Q. Zhang, B. Zhao, Q. Wang, C. Zhu, Z. Liu, Y. Zheng, J. Su, Y. Gao, *Nano Energy* **2022**, *93*, 106896.
